# Overexpression of lncRNA HOXA-AS2 promotes the progression of oral squamous cell carcinoma by mediating SNX5 expression

**DOI:** 10.1186/s12860-022-00457-y

**Published:** 2022-12-17

**Authors:** Zhangyi Li

**Affiliations:** Department of Stomatology, the Fifth Central Hospital of Tianjin, No. 41, Zhejiang Road, Tanggu, Binhai New District, 300450 Tianjin, China

**Keywords:** Oral squamous cell carcinoma, HOXA-AS2, SNX5, miR-520c-3p, Autophagy

## Abstract

**Background:**

Oral squamous cell carcinoma (OSCC) is one of the most common head and neck cancers. Long non-coding RNA HOXA-AS2 (lncRNA HOXA-AS2) have been extensively studied in various cancers. However, the expression and function of HOXA-AS2 in OSCC still remain unknown. The aim of this study is to investigate the roles of HOXA-AS2 in OSCC.

**Methods:**

OSCC tissues and adjacent normal tissues were obtained from OSCC patients. RT-qPCR and Western blot assays were used to detect the expression of target genes in OSCC tissues or cells. Cells proliferation, migration and invasion were detected by CCK-8 and transwell assays, respectively. The target gene of HOXA-AS2 was confirmed by dual-luciferase reporter gene assay.

**Results:**

We found that HOXA-AS2 expression was remarkably upregulated in OSCC tissues and cell lines. The downregulation of HOXA-AS2 inhibited cells proliferation, migration and invasion. Our bioinformatics analysis found that HOXA-AS2 can target miR-520c-3p, which was confirmed by dual-luciferase reporter gene assay. The expression of HOXA-AS2 was found to be negatively associated with miR-520c-3p in OSCC tissues. Moreover, sorting nexin 5 (SNX5), a downstream target of miR-520c-3p, was inhibited by miR-520c-3p overexpression. SNX5 was also increased in OSCC tissues and cell lines. Additionally, we found that the higher expression of SNX5 was strongly associated with the tumor grade of OSCC patients in Oncomine database. Most importantly, the knockdown of HOXA-AS2 induced cells apoptosis by promoting autophagy by regulating SNX5.

**Conclusion:**

HOXA-AS2 served an oncogene and promoted OSCC progression via the miR-520c-3p/SNX5 axis. Thus, HOXA-AS2 may be a new biomarker for diagnosis and treatment of OSCC.

**Supplementary Information:**

The online version contains supplementary material available at 10.1186/s12860-022-00457-y.

## Introduction

OSCC represents one of the most common head and neck cancers with a high mortality rate due to lack of early diagnosis markers, advanced treatment and understanding of the pathogenic mechanisms [1, 2]. At present, the main treatments for OSCC include surgery, radio- and chemo-therapeutics. Although critical improvement for OSCC treatments have been made in over the past few years, the overall 5-year survival rate of OSCC patients is less than 60% [3]. Therefore, the prerequisite for the better diagnosis and treatments of OSCC is to further explore the pathogenic mechanisms underlying OSCC.

In recent years, long noncoding RNAs (lncRNAs), with over 200 nucleotides in length and without protein-coding function, have been extensively studied in different types of cancers [4]. LncRNAs regulate the progression of cancers at translation, epigenetic, transcriptional or post-transcriptional levels [5]. Accumulating evidence has reported that lncRNAs play its role in tumorigenesis through sponging microRNAs [6–8]. For instance, lncRNA-RMRP expression is up-regulated in degenerated nucleus pulposus tissues, it promotes nucleus pulposus cell proliferation via regulating miR-206 expression [9]. SNHG15 promotes the progression of colorectal cancer by regulating cell proliferation, invasion and drug resistance by interacting with AIF [10]. Long non-coding RNA TP73-AS1 accelerates osteosarcoma cell proliferation and invasion through targeting miR-142 [11]. Some studies indicated that the expression of HOXA-AS2 was strikingly upregulated in many types of cancers, such as in non-small cell lung cancer, bladder cancer and pancreatic cancer [12–15]. In addition, it was found that HOXA-AS2 promoted OSCC progression by upregulating EZH2 [16] or inhibiting miR-567 expression [17]. But, a better understanding of the roles of HOXA-AS2 in OSCC remains to be further studied.

In this study, we found that HOXA-AS2 expression is highly increased in OSCC tissues and cell lines. Silencing HOXA-AS2 inhibited cell proliferation, migration, invasion autophagy, and induced cell apoptosis. Mechanistically, HOXA-AS2 regulated OSCC cells growth through the miR-520c-3p/SNX5 pathway.

## Results

### HOXA-AS2 is upregulated in OSCC

**Fig. 1:**
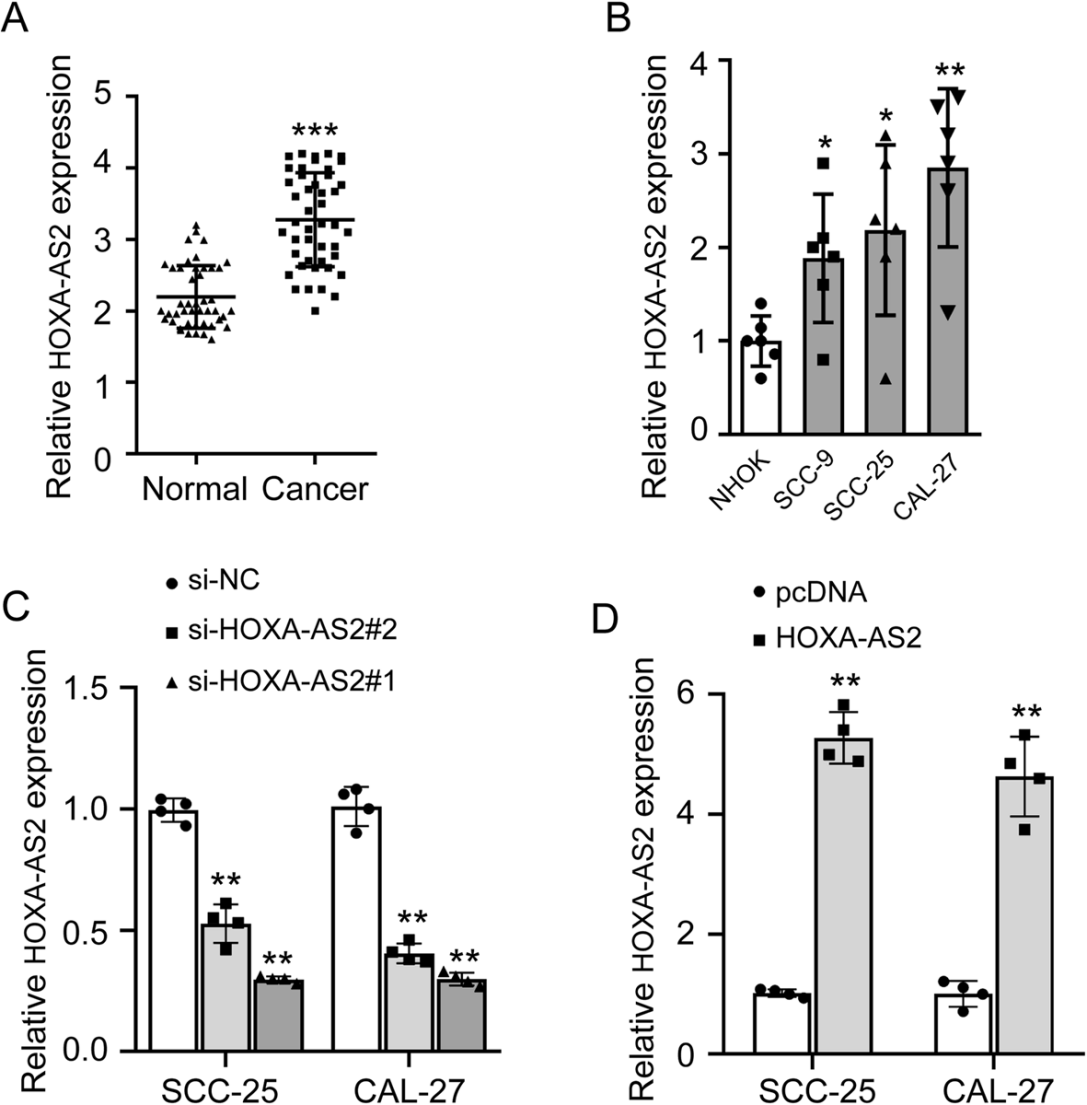
HOXA-AS2 is highly expressed in the OSCC. **A** HOXA-AS2 expression levels were detected in OSCC cancer tissues and their adjacent normal tissues by RT-qPCR. **B** HOXA-AS2 expression were detected in normal human oral keratinocyte (NHOK) and OSCC cell lines (CAL-27, SCC-9, SCC-25) by RT-qPCR. **C** The SCC-25 and CAL-27 were transfected with siRNA to knock down HOXA-AS2, and knockdown efficiency was confirmed by RT-qPCR. **D** Overexpression results of HOXA-AS2 were detected by RT-qPCR in SCC-25 and CAL-27 cells transfected with overexpression plasmids of HOXA-AS2. The experiment was repeated at least three times. *P < 0.05 and **P < 0.01

In order to investigate the role of HOXA-AS2 in OSCC, we first analyzed the expression of HOXA-AS2 in 45 cases of OSCC tissues and their adjacent normal tissues by RT-qPCR. The result showed that HOXA-AS2 was highly expressed in OSCC tissues compared with adjacent normal tissues (Fig. 1A). By analyzing the clinical characteristics of OSCC patients, we found that the expression of HOXA-AS2 is positively correlated with the TNM stage, but negatively correlated with the degree of differentiation of tumor tissues of OSCC patients (Table 1). We also detected the endogenous levels of HOXA-AS2 in NHOK and OSCC cell lines (SCC-9, SCC-25, CAL-27) using RT-qPCR. The expression of HOXA-AS2 was significantly upregulated in OSCC cells compared to NHOK cells (Fig. 1B). SCC-25 and CAL-27 cells, the two OSCC cell lines with the higher expression of HOXA-AS2, were selected for the following assays. To explore the effect of HOXA-AS2 in OSCC cells, we used two small interfering RNA (siRNA) to knock down the expression of HOXA-AS2. We detected the knockdown efficiency of two siRNAs (si-HOXA-AS2#1 and si-HOXA-AS2#2) in SCC-25 and CAL-27 cells. si-HOXA-AS2#1 decreased HOXA-AS2 by about 3.4 fold in both cells, while si-HOXA-AS2#2 decreased HOXA-AS2 by about 1.9 and 2.4 fold in both cells, respectively (Fig. 1C). Thus, si-HOXA-AS2#1 was selected for all subsequent experiments for its more pronounced knockdown efficiency. Additionally, we increased the expression of HOXA-AS2 using overexpression plasmids in SCC-25 and CAL-27 cells (Fig. 1D).

**Table 1:** The HOXA-AS2 expression and its relationship with clinicopathological characteristics of OSCC patients (*n* = 45)

Characteristics	Total	HOXA-AS2 expression		*p* value
		low	high	
Age				
40	13	7	6	
≥40	32	13	19	0.419
Gender				
Male	20	11	9	
Female	25	9	16	0.202
TNM Stage				
I+II	21	13	8	
III+IV	24	7	17	0.027*
Differentiation				
Poor	19	5	14	
Well	26	15	11	0.036*

Data were analyzed with Chi-square test, *p < 0.05 was considered statistically significant.

### Knockdown of HOXA-AS2 suppressed OSCC cells proliferation, migration, invasion

**Fig. 2:**
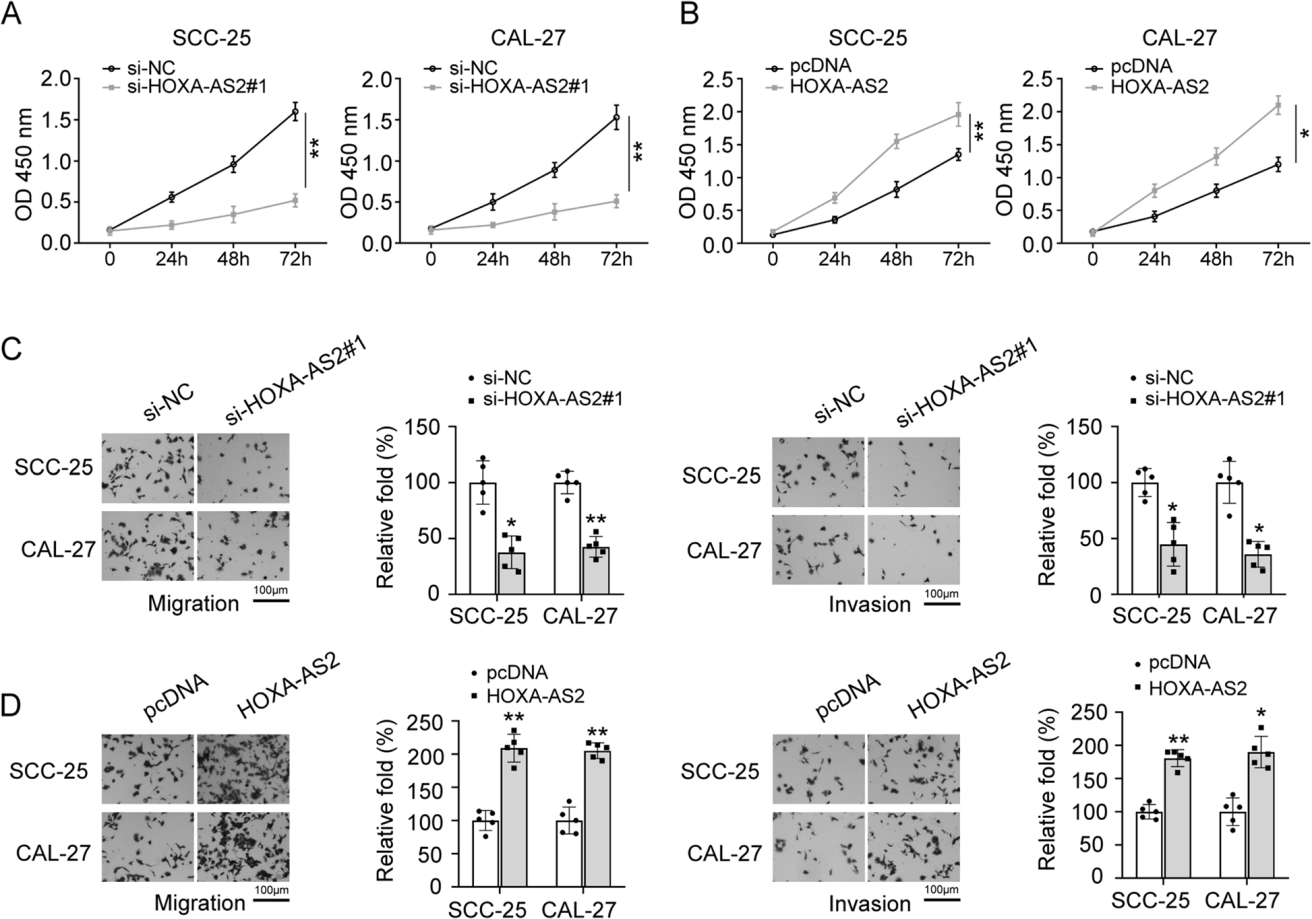
The effect of HOXA-AS2 on OSCC cells proliferation, migration and invasion. **A**, **B** SCC-25 and CAL-27 cells were transfected with si-HOXA-AS2#1 or overexpression plasmids of HOXA-AS2, CCK8 assay was conducted to evaluate the effect of altering the HOXA-AS2 expression on cell proliferation. **C**, **D** Cell migration and invasion capacities were detected by transwell assays in SCC-25 and CAL-27 cells transfected with si-HOXA-AS2#1 or overexpression plasmids of HOXA-AS2, respectively. The experiment was repeated at least three times. *P < 0.05 and **P < 0.01

To explore the roles of HOXA-AS2 in OSCC cells, SCC-25 and CAL-27 cells were transfected with si-HOXA-AS2#1. Then, cells proliferation, migration, invasion capacities were measured. The results showed that cells proliferation was markedly inhibited for the knockdown of HOXA-AS2 (Fig. 2A). As expected, the cells proliferation was obviously improved after HOXA-AS2 overexpression (Fig. 2B). Furthermore, we also analyzed the migration and invasion abilities of OSCC cells using transwell assays. As displayed in Fig. 2C, knockdown of HOXA-AS2 can inhibit migration and invasion abilities of OSCC cells, while overexpression of HOXA-AS2 promoted cells migration and invasion (Fig. 2D). These findings indicated that knockdown of HOXA-AS2 significantly inhibited the proliferation, migration, invasion of OSCC cells.

### HOXA-AS2 negatively targets miR-520c-3p in OSCC

**Fig. 3:**
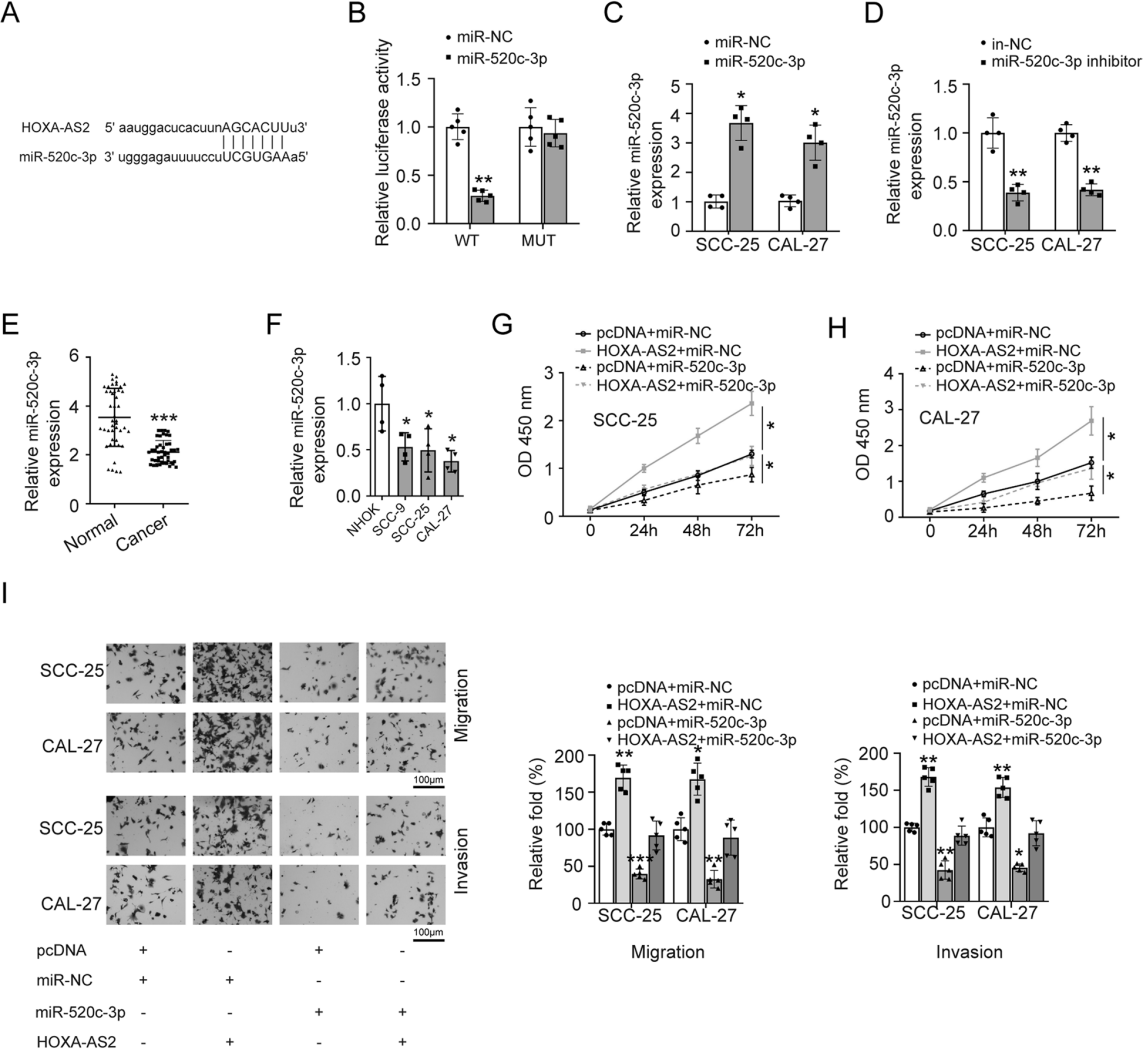
miR-520c-3p expression was negatively regulated by HOXA-AS2. **A** The binding site between HOXA-AS2 and miR-520c-3p was predicted from starBase database. **B** The role of miR-520c-3p on cell luciferase activity of HOXA-AS2-WT and HOXA-AS2-MUT reporter systems was measured by luciferase reporter assay. **C**, **D** SCC-25 and CAL-27 cells transfected with miR-520c-3p mimic or inhibitor, and the expression levels of miR-520c-3p were detected by RT-qPCR. **E** The expression of miR-520c-3p was measured in OSCC cancer tissues and their adjacent normal tissues by RT-qPCR. **F** miR-520c-3p expression was detected in normal human oral keratinocyte (NHOK) and OSCC cell lines (CAL-27, SCC-9, SCC-25) by RT-qPCR. SCC-25 and CAL-27 cells were respectively transfected with pcDNA plus miR-NC, HOXA-AS2 plus miR-NC, pcDNA plus miR-520c-3p mimic, or HOXA-AS2 plus miR-520c-3p mimic, **G**, **H** cell proliferation was detected by CCK8 assay, **I** cell migration and invasion abilities were detected by transwell assays. The experiment was repeated at least three times. *P < 0.05, **P < 0.01 and ***P < 0.001

Accumulating reports indicated that lncRNAs can competitively target miRNAs to affect the development of cancers [18]. Thus, we predicted the potential targets of HOXA-AS2 using starBase database. The results showed that HOXA-AS2 potentially bind to miR-520c-3p (Fig. 3A). Dual-luciferase reporter gene assay determined that the luciferase activity was apparently inhibited by miR-520c-3p mimic in HOXA-AS2-WT transfection group instead of HOXA-AS2-MUT group (Fig. 3B). To further explore the role of miR-520c-3p in OSCC, SCC-25 and CAL-27 cells were transfected with miR-520c-3p mimic or inhibitor, and miR-520c-3p expression levels were evaluated (Fig. 3C, D). Further, we found that the expression levels of miR-520c-3p in OSCC tissues and OSCC cell lines was significantly lower than that of normal control group (Fig. 3E, F). To explore the role of HOXA-AS2/miR-520c-3p in the development of OSCC, OSCC cells were co-transfected with HOXA-AS2 and miR-520c-3p mimic. The CCK-8 assay demonstrated that the overexpression of HOXA-AS2 obviously rescued the inhibition of miR-520c-3p overexpression on cell proliferation (Fig. 3G, H). In addition, we also found that miR-520c-3p overexpression inhibited the migration and invasion of OSCC cells, which was reversed by the overexpression of HOXA-AS2 (Fig. 3I). These results revealed that HOXA-AS2 could sponge miR-520c-3p to promote the development of OSCC.

### The target gene prediction and expression analysis of miR-520c-3p in OSCC

**Fig. 4:**
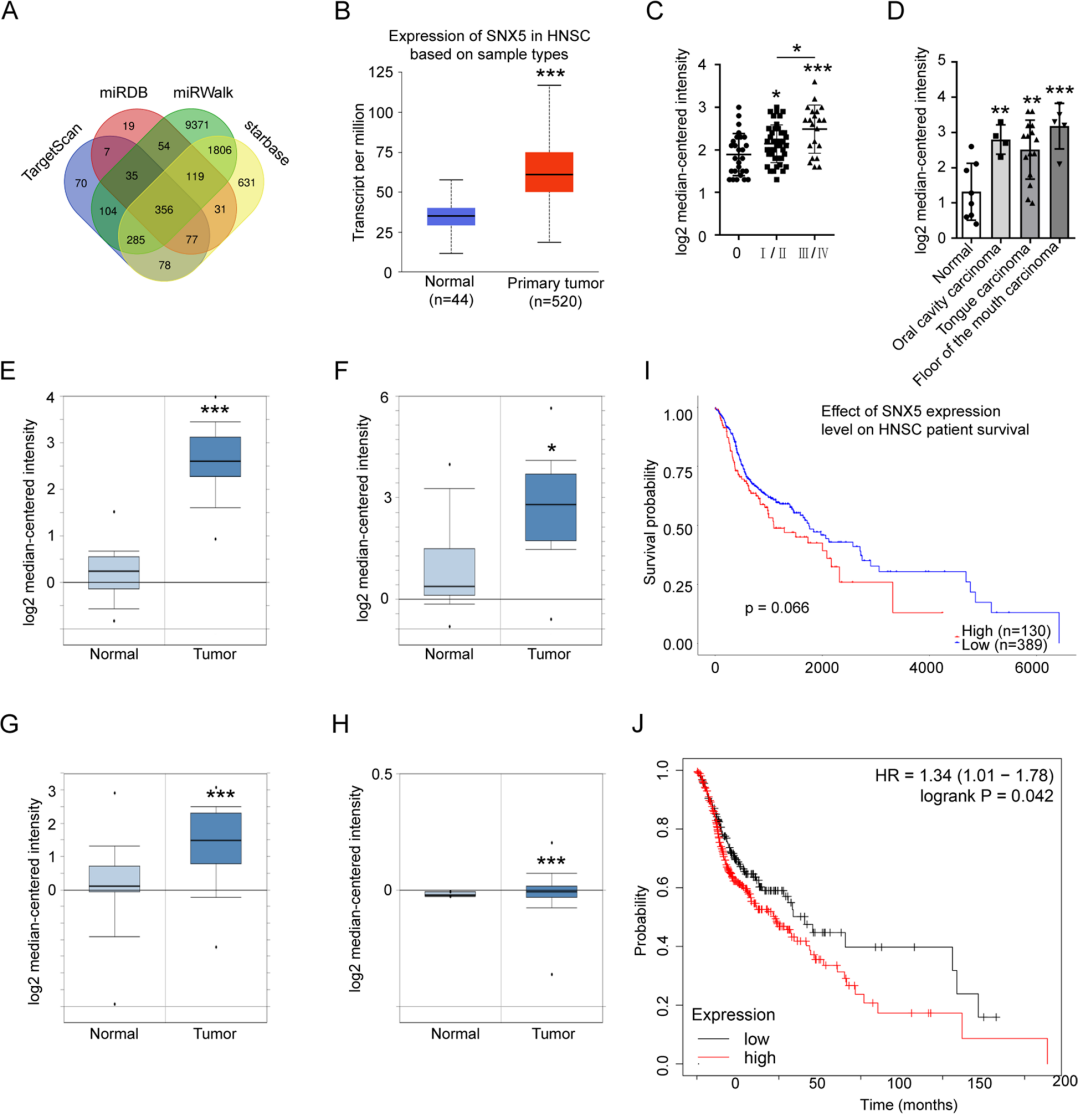
The target genes prediction and analysis of miR-520c-3p. **A** The target genes of miR-520c-3p were predicted by miRDB, miRWalk, TargetScan and starbase databases. **B** The expression levels of SNX5 in HNSC was obtained from UALCAN database. **C** SNX5 expression level was positively related to the different advanced pathological stages of OSCC in Oncomine database. 0: benign tumor, I/II: tumor grade I/II, III/IV: tumor grade III/IV. **D** SNX5 expression was increased in oral cancers in Pyeon Multi-cancer datasets in Oncomine database. 1: normal, 2: oral cavity carcinoma, 3: tongue carcinoma, 4: floor of the mouth carcinoma. **E-H** SNX5 expression levels were analyzed in oral cancers in Oncomine database (**E-H**). **E** Oral Cavity Squamous Cell Carcinoma in Peng Head-Neck. **F** Oral Cavity Squamous Cell Carcinoma epithelia in Toruner Head-Neck. **G** Tongue Squamous Cell Carcinoma in Ye Head-Neck. **H** Oral Cavity Squamous Cell Carcinoma in Peng Head-Neck 2. **I**, **J** High expression of SNX5 was associated with poor prognosis of OSCC patients in UALCAN and Kaplan Meier Plotter databases, respectively.*P < 0.05, **P < 0.01 and ***P < 0.001

To further explore the potential regulatory mechanism of miR-520c-3p in OSCC, we predicted the target genes of miR-520c-3p by using miRDB, miRWalk, TargetScan and starbase databases (Fig. 4A). Because OSCC is the most common head and neck squamous cell carcinoma (HNSC). So, we first to explore whether SNX5 was related to HNSC by the using UALCAN database. We found that the expression level of SNX5 was highly increased in HNSC tumor tissues compared with normal tissues (Fig. 4B). Furthermore, the higher mRNA expression level of SNX5 was positively related to the different advanced pathological stages of OSCC (Fig. 4C), and was found in oral cancer from Oncomine database (Fig. 4D). Meanwhile, SNX5 was significantly elevated in oral-related cancers (Fig. 4E-H). Kaplan-Meier survival analysis showed that the survival time of HNSC patients with upregulated SNX5 expression was distinctly shorter than that of patients with low expression (Fig. 4I, J). Thus, these results showed that SNX5 may play key a carcinogenesis in OSCC development.

### Overexpression of SNX5 promotes OSCC development

**Fig. 5:**
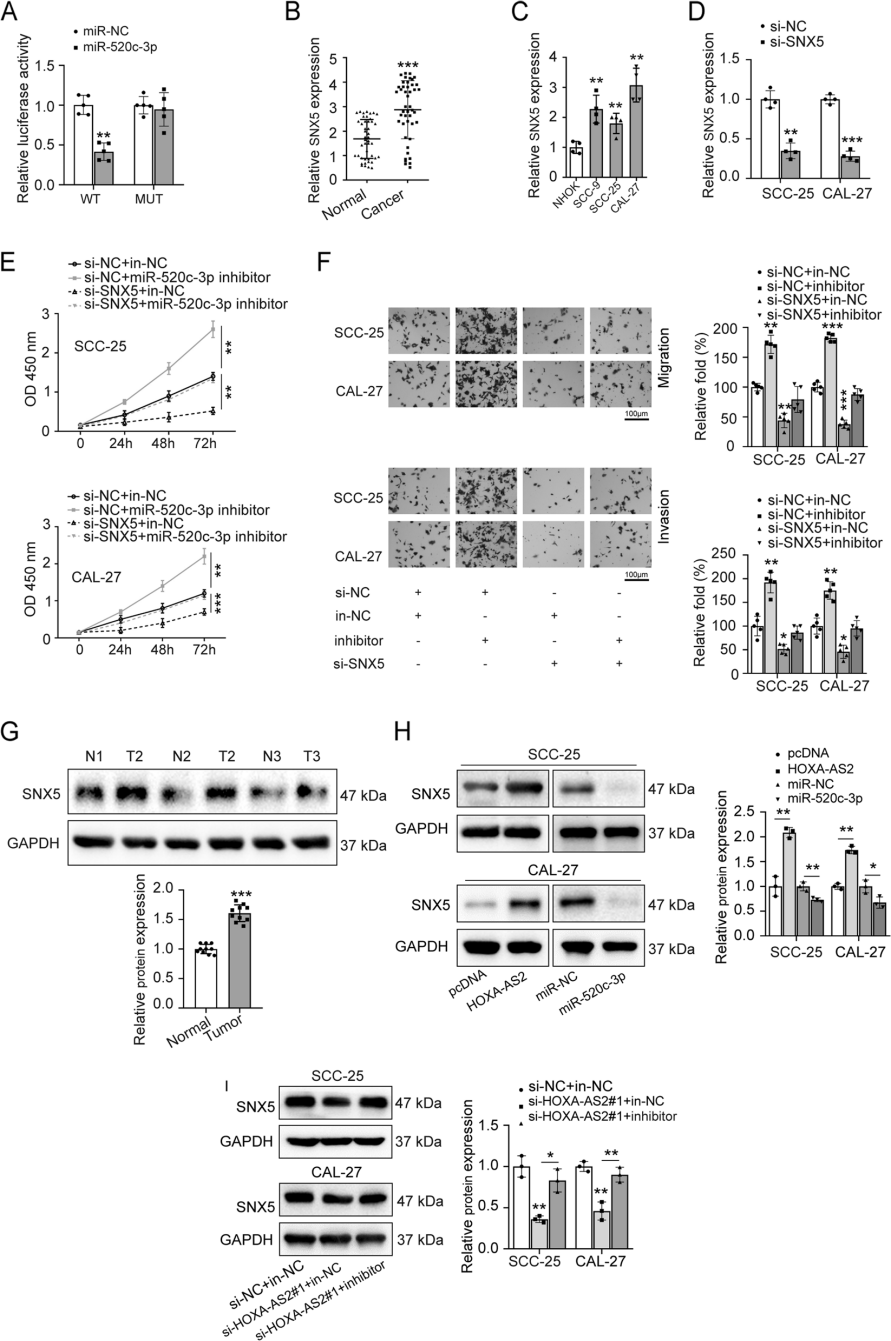
SNX5 is a target of miR-520c-3p. A The role of miR-520c-3p on cell luciferase activity of SNX5-WT and SNX5-MUT reporter systems was measured by luciferase reporter assay. **B**, **C** The expression levels of SNX5 in OSCC tissues and cells were detected by RT-qPCR. **D** The knockdown level of SNX5 was confirmed in SCC-25 and CAL-27 cells by RT-qPCR. SCC-25 and CAL-27 cells were respectively transfected with si-NC plus in-NC, si-NC plus miR-520c-3p inhibitor, si-SNX5 plus in-NC, or si-SNX5 plus miR-520c-3p inhibitor, **E** cell proliferation was detected by CCK8 assay, **F** cell migration and invasion abilities were detected by transwell assays. **G** The SNX5 protein expression was explored in OSCC tissues and their adjacent normal tissues by western blot. **H** The effect of overexpression of HOXA-AS2 or miR-520c-3p on SNX5 protein expression was evaluated by western blot. **I** SCC-25 and CAL-27 cells were respectively transfected with si-NC plus in-NC, si-HOXA-AS2#1 plus in-NC, or si-HOXA-AS2#1 plus miR-520c-3p inhibitor, then SNX5 protein expression was detected by western blot. The experiment was repeated at least three times. *P < 0.05, **P < 0.01, and ***P < 0.001

The luciferase assay demonstrated that miR-520c-3p targeted the 3’-UTR region of SNX5 (Fig. 5A), which showed that SNX5 is target gene of miR-520c-3p. In addition, the expression levels of SNX5 were significantly increased in OSCC cancer tissues and cell lines (Fig. 5B, C). To reveal the effect of SNX5 on cell proliferation, migration and invasion, we knocked down the expression of SNX5 with siRNA in SCC-25 and CAL-27 cells (Fig. 5D). Then, SCC-25 and CAL-27 cells were transfected with si-SNX5 and miR-520c-3p inhibitor to reveal the role of miR-520c-3p/SNX5 axis in OSCC. CCK8 assay results indicated that knockdown of SNX5 decreased cell proliferation ability, which was reversed by miR-520c-3p inhibitor (Fig. 5E). In addition, the knockdown of SNX5 remarkably inhibited the migration of OSCC cells, while miR-520c-3p inhibitor rescued the inhibition of knockdown of SNX5 on cell migration (Fig. 5F). Similar results were also found in cell invasion assay (Fig. 5F). Moreover, the higher protein expression of SNX5 was found in OSCC tissues (Fig. 5G). This fact further indicates that SNX5 may play a carcinogenic role in OSCC. Next, to study the regulation of HOXA-AS2 and miR-520c-3p on SNX5 expression. We determined that the overexpression of HOXA-AS2 and miR-520c-3p respectively promoted or inhibited the protein expression of SNX5 in OSCC cells (Fig. 5H). Furthermore, Inhibition of miR-520c-3p ameliorated the inhibitory effect of knockdown of HOXA-AS2 on SNX5 protein expression (Fig. 5I). It is indicated that HOXA-AS2 can competitively target miR-520c-3p and then regulate the protein expression of SNX5 by acting as a ceRNA. In a word, HOXA-AS2/miR-520c-3p play its promotion on OSCC development by upregulating SNX5 expression.

### Silencing of HOXA-AS2 induces apoptosis by regulating autophagy in a SNX5-dependent manner in OSCC cells

**Fig. 6:**
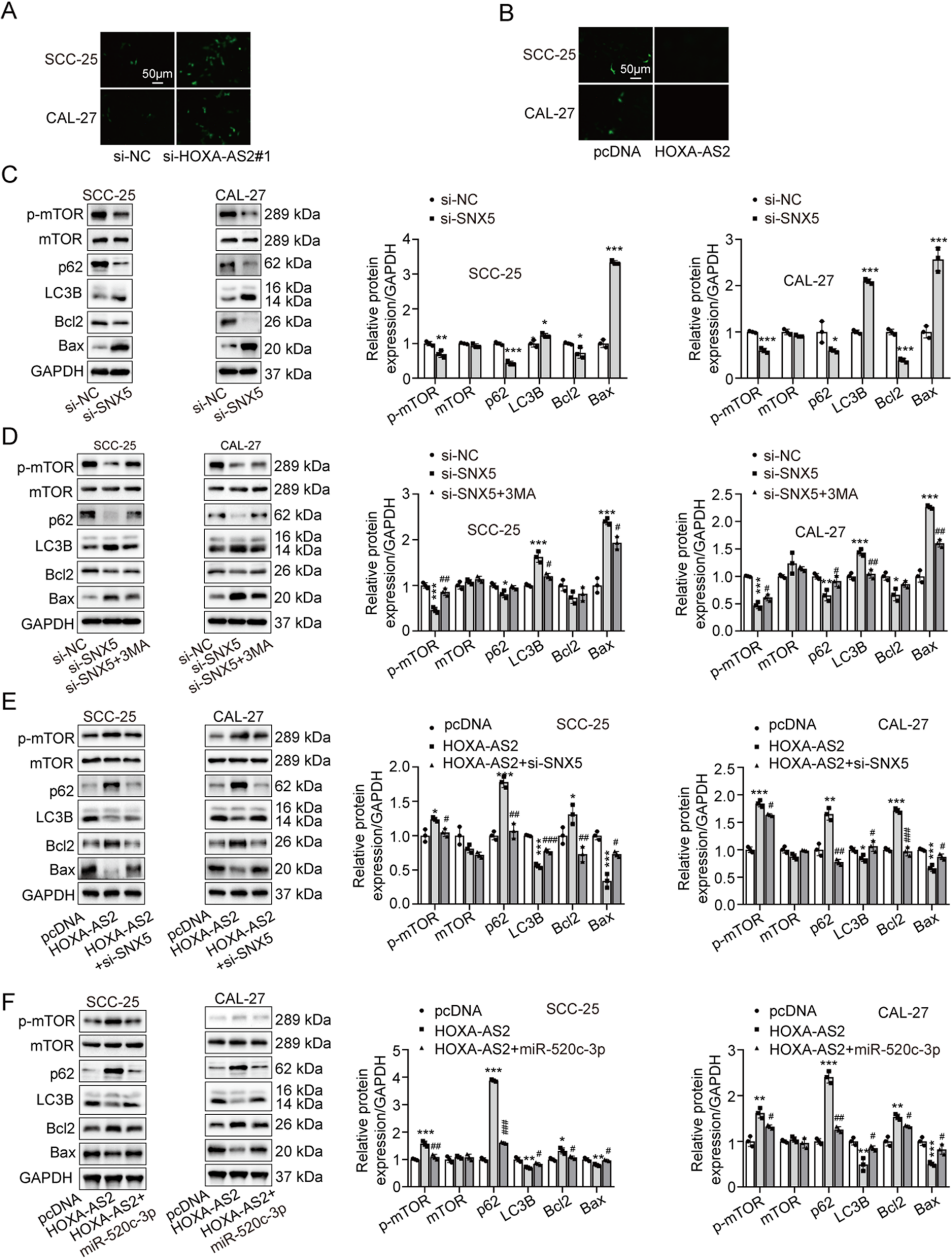
HOXA-AS2 regulates autophagy-mediated apoptosis through SNX5. **A**, **B** SCC-25 and CAL-27 cells were transfected with si-NC, si-HOXA-AS2#1, pcDNA, or HOXA-AS2, cell apoptosis was evaluated using TUNEL assay. Western blot was used to detect the protein expression levels of p-mTOR, mTOR, p62, LC3B, Bcl2, Bax in SCC-25 and CAL-27 cells with different transfections or treatment. **C** SCC-25 and CAL-27 cells were transfected with si-NC or si-SNX5. **D** SCC-25 and CAL-27 cells were transfected with si-NC, si-SNX5, or si-SNX5 plus treatment of 3MA. **E** SCC-25 and CAL-27 cells were transfected with pcDNA, HOXA-AS2, or HOXA-AS2 plus si-SNX5. **F** SCC-25 and CAL-27 cells were transfected with pcDNA, HOXA-AS2, or HOXA-AS2 plus miR-520c-3p mimic. The experiment was repeated at least three times. *P < 0.05, **P < 0.01 and ***P < 0.001 compared with si-NC or pcDNA; #*P* < 0.05, ##*P* < 0.01 and ###*P* < 0.001 compared with HOXA-AS2

Autophagy plays a key role in OSCC by regulating cell apoptosis [19, 20]. In addition, Zhou et. al reported that SNX5 increased ERK1/2 expression [21]. As inhibitor of autophagy, ERK1/2 can restrain autophagy by activating the mTOR signaling pathway [22]. Thus, we investigated the relationship between autophagy and apoptosis in OSCC cells, and further explored whether HOXA-AS2 participated in apoptosis and autophagy in a SNX5-dependent manner. We first knocked down or overexpressed HOXA-AS2 in OSCC cells and then examined cell apoptosis using TUNEL assay. The results showed that the knockdown of HOXA-AS2 promoted cell apoptosis (Fig. 6A), while cell apoptosis was inhibited by the overexpression of HOXA-AS2 (Fig. 6B). Next, we found that the silencing of SNX5 promoted the expression of Bax and LC3B, while the expression of p-mTOR, Bcl2 and p62 were inhibited (Fig. 6C). These results showed that the silencing of SNX5 induced cell apoptosis and promoted autophagy. To reveal whether activated autophagy promotes apoptosis of OSCC cells, we suppressed autophagy with 3MA (an autophagy inhibitor) in cells transfected si-SNX5. Western blot analysis indicated that 3MA greatly reversed the apoptosis and autophagy induced by knockdown SNX5 (Fig. 6D). We further investigated whether HOXA-AS2 regulates autophagy and apoptosis through the miR-520c-3p/SNX5 axis. After different transfection, results showed that the overexpression of HOXA-AS2 significantly suppressed cell autophagy and apoptosis (Fig. 6E, F). However, inhibition of SNX5 or overexpression of miR-520c-3p could improve the inhibition of overexpression of HOXA-AS2 on cell autophagy and apoptosis (Fig. 6E, F). In conclusion, these results suggested that HOXA-AS2/miR-520c-3p/SNX5 axis affected the OSCC cell apoptosis by regulating autophagy.

## Discussion

OSCC is one of the most common malignancies worldwide [23]. Although the enormous advance was achieved in the diagnosis and treatment for OSCC in the past decades. The occurrence of OSCC is still at a higher level [24]. Thus, the underlying pathogenic mechanism of OSCC needs to be further explored.

Increasing evidences have indicated that lncRNAs play critical roles in tumorigenesis, cancer progression and metastasis [25–28]. Thus, the roles of lncRNAs in OSCC have been largely revealed in recent years [29, 30]. LncRNA HOXA-AS2 has been reported to be upregulated and associated with various cancers progression. For instance, Han et al found that HOXA-AS2 was up-regulated in osteosarcoma tissues, overexpression of HOXA-AS2 promotes cells migration and invasion [31]. Xu et al showed that HOXA-AS2 was highly expressed in papillary thyroid cancer tissues and cell lines, the knockdown of HOXA-AS2 inhibited papillary thyroid cancer progression by mediating miR-15a-5p/HOXA3 axis [32]. Yan et al also indicated that the increased HOXA-AS2 expression was associated with advanced tumor stage by regulating RND3 expression [33]. In agreement with these findings, we found that HOXA-AS2 was highly expressed in OSCC tissues and cell lines. The knockdown of HOXA-AS2 suppressed the cells proliferation, migration and invasion, and induced apoptosis.

Many studies have reported that lncRNAs could sponge to miRNAs and regulate target genes expression of miRNAs [34]. Thus, to further explore the molecular mechanism of HOXA-AS2 in OSCC progression, we first predicted and confirmed that miR-520c-3p was a target of HOXA-AS2. The anti-tumour role of miR-520c-3p have been found in some cancers. For instance, in lung adenocarcinoma, miR-520c-3p have a lower expression level and regulates its targets AKT1 and AKT2 expression [35]. Increased miR-520c-3p expression level inhibited cell proliferation and initiated premature senescence in HeLa and DLBCL cells by directly targeting eIF4GII [36]. In osteosarcoma cells, lncRNA HOXA-AS2 promotes cells migration and invasion by inhibiting miR-520c-3p expression [31]. In this study, we also found a lower expression levels of miR-520c-3p in OSCC tissues and cell lines. Overexpression of miR-520c-3p obviously suppressed the cells growth, migration, and invasion. However, the inhibition of miR-520c-3p on the malignant properties of OSCC cells was restored by overexpressing HOXA-AS2.

In further studies, we determined SNX5 as a candidate target gene of miR-520c-3p. SNX5 was reported as a tumor promoter in a variety of cancers. For instance, the expression of SNX5 was upregulated in HCC, which was associated with poor prognosis of HCC patients and promoted cells proliferation, migration, invasion and metastasis [21]. In head and neck squamous cell carcinoma (HNSCC), SNX5 was also significantly elevated [37]. In present study, we indicated that SNX5 expression was significantly increased in oral-related cancer and positively related to advanced pathological stagesof OSCC in Oncomine database. Consistently, we found that SNX5 expression was upregulated in OSCC tissues and cell lines by RT-qPCR. miR-520c-3p inhibitor reversed tumor suppressor effect of knockdown of SNX5. Moreover, we also found that HOXA-AS2 regulates autophagy-mediated apoptosis in a miR-520c-3p/SNX5-dependent manner.

In conclusion, we found that HOXA-AS2 functioned as an oncogene in OSCC. The overexpression of HOXA-AS2 accelerated the progression of OSCC by promoting cells proliferation, migration and invasion, and inhibiting autophagy-mediated apoptosis via miR-520c-3p/SNX5 axis. Thus, our findings may provided a novel prognostic marker for the treatment of OSCC.

## Methods

### Patient tissue samples

OSCC tissues and their adjacent normal tissues were obtained from patients at the Fifth Central Hospital of Tianjin. The informed consent was obtained from all subjects and/or their legal guardian(s). OSCC tissues were snap-frozen in liquid nitrogen and then stored at -80℃. The clinicopathological characteristics of OSCC patients are shown in Table 1. This study was approved by the Ethics Committee of the Fifth Central Hospital of Tianjin.

### Cell culture

The normal human oral keratinocyte (NHOK), OSCC cell lines (CAL-27, SCC-9, SCC-25) and HEK-293T cells were obtained from Cell Bank of the Chinese Academy of Sciences (Shanghai, China). All cells were cultured with DMEM (Gibco, USA) medium containing 10% fetal bovine serum (FBS) (Invitrogen, USA) and 1% penicillin-streptomycin in humidified air with 5% CO_2_ at 37 °C. Passaging is required when the cell confluence reaches 80%-90%.

### Cell transfection

The small interference RNA (si-RNA) specifically targeting HOXA-AS2 (si-HOXA-AS2) and its negative control, pcDNA3.1-HOXA-AS2 overexpression plasmid (HOXA-AS2) and empty vector (pcDNA), miR-520c-3p mimic, miR-520c-3p inhibitor and their respective negative control miR-NC or in-NC were synthesized by Shanghai GenePharma (GenePharma, China). Cells were transfected using Lipofectamine 2000 reagent (Invitrogen) following the manufacturer’s instructions.

### RNA isolation and RT-qPCR

Total RNA was isolated from OSCC tissues or cells using the Trizol reagent (Invitrogen) according to the instructions. cDNA was generated using High-Capacity cDNA Reverse Transcription Kit (Thermo, USA). Real-time qPCR analysis was was carried out with Maxima SYBR Green qPCR Master Mix (Fermentas) using an ABI 7500 fast system (Thermo, USA). Relative expression of genes was calculated using the 2^−ΔΔCT^ method.

### CCK-8 assay

SCC-25 and CAL-27 cells were seeded in 96-well plates at a density of 2×10^3^ cells/100 μl per well. CCK8 assay was conducted at 24, 48, and 72 h. Briefly, 10 μl of CCK-8 solution was added to each well and incubated with cells for 4 h at 37 °C. Then, the optical density was determined using a microplate reader (Bio-Rad, Hercules, CA, USA) at 450 nm. Cell proliferation curves were generated following the manufacturer’s instructions.

### Transwell assay

A total of 5×10^4^ SCC-25 or CAL-27 cells were resuspended in serum-free DMEM medium and seeded into the upper inserts of transwell inserts with a pore size of 8 μm. DMEM medium containing 10% FBS was added into the lower inserts. After incubation for 24 h, cells were fixed with 4% paraformaldehyde and stained with 0.1% crystal violet for 10 minutes at room temperature. The number of cells was counted using a microscope. For cell invasion assay, all procedures were the same as cell migration assay except that the upper chambers of transwel were pre-coated with matrigel matrix (BD Biosciences, USA).

### Western blot

Total protein was isolated from OSCC tissues or cells using Radio-Immunoprecipitation Assay (RIPA) lysis buffer (Beyotime, Beijing, China). Protein samples were separated on sodium dodecyl sulfate-polyacrylamide gel and transferred to a polyvinylidene fluoride membrane (PVDF, Schwalbach, Germany). The membrane was blocked by 5% nonfat milk for 2 h at room temperature and incubated with the following primary antibodies at 4°C overnight: anti-Bcl2 (1:1000, 4223, Cell signaling technology, USA), anti-Bax (1:1000, 2772, Cell signaling technology, USA), anti-SNX5 (1:1000, ab180520, Abcam, USA), anti-p-S2448-mTOR (1:2000, ab109268, Abcam, USA), anti-mTOR (1:2000, ab32028, Abcam, USA), anti-p62 (1:2000, ab109012, Abcam, USA), anti-LC3B (1:2000, ab192890, Abcam, USA), as well as anti-GAPDH (1:10 000, 8884, Cell signaling technology, USA). Detection of mTOR phosphorylation requires fresh protein samples. Then the membrane was incubated with horseradish peroxidase (HRP)-labeled secondary antibody for 1 h at room temperature. The bands were detected using an enhanced chemiluminescence detection system. GAPDH is the loading control.

### Tunel assay

The TUNEL assay was used to detect the cell apoptosis using the one-step TUNEL Apoptosis Detection Kit (Beyotime, China) following the manufacturer’s instructions. Briefly. OSCC cells were washed once with PBS and fixed with 4% paraformaldehyde for 30 min. Next, cells were incubated with 0.3% Triton X-100 for 5 min, followed by TUNEL staining solution for an additional 60 min. TUNEL-positive cells were imaged using an fluorescence microscope (Olympus, Japan).

### Dual-luciferase reporter assay

Complete fragments of the 3ʹ-untranslated region (3ʹ-UTR) of HOXA-AS2 and SNX5 were generated by PCR amplification and cloned into the downstream the luciferase gene to obtain HOXA-AS2-wild type (HOXA-AS2-WT) and SNX5-wild type (SNX5-WT) plasmids. And the potential binding sites of HOXA-AS2 and SNX5 on miR-520c-3p were mutated to construct HOXA-AS2-MUT and SNX5-MUT plasmids. Then, HEK-293T cells were co-transfected with HOXA-AS2-WT, HOXA-AS2-MUT, miR-520c-3p mimic or mi-NC using Lipofectamine2000. SNX5-WT or SNX5-MUT were co-transfected with miR-520c-3p mimic or mi-NC into HEK-293T cells, respectively. Forty-eight hours post transfection, cells were harvested and luciferase activity was measured using Dual-luciferase Reporter Assay System (Promega) following the manufacturer’s instructions.

### Bioinformatic analysis

The potential target of HOXA-AS2 was predicted using starBase database (http://starbase.sysu.edu.cn). We predicted the target genes of miR-520c-3p using miRDB (http://mirdb.org/), miRWalk (http://mirwalk.umm.uni-heidelberg.de/), TargetScan (http://www.targetscan.org/vert_71/) and starBase databases. The relationship between SNX5 and survival of HNSC patients was analyzed using UALCAN (http://ualcan.path.uab.edu/) and Kaplan Meier Plotter databases (http://kmplot.com/analysis/). mRNA expression level of SNX5 was analyzed in OSCC using Oncomine database (https://www.oncomine.org/resource/login.html).

### Statistical analysis

Data presented as mean ± standard deviation (SD). All data were obtained from at least three separate experiments and analyzed by SPSS 16.0 and GraphPad Prism 5.0 software software. The difference of gene expression between normal and cancer tissues were analyzed using paired t-tests. Otherwise, unpaired t-tests was used to compare the differences between the other two groups. Comparison of multiple groups were estimated with one-way ANOVA followed by Tukey's post hoc test. All data represent three independent experiments. *p* < 0.05 was considered statistically significant.

## Supplementary Information

**Additional file 1.** Statement for experiments involving human participants.

**Additional file 2.** Supplement Original migration and invasion images.

**Additional file 3.** Supplementary Original western blot images.

## Data Availability

The datasets used and/or analyzed during the current study are available from the corresponding author on reasonable request.
