# Supplementary material for: Overexpression of lncRNA HOXA-AS2 promotes the progression of oral squamous cell carcinoma by mediating SNX5 expression
Source: BMC Mol Cell Biol. 2022 Dec 17;23:59. doi: 10.1186/s12860-022-00457-y (PMC9759889; doi:10.1186/s12860-022-00457-y)
Supplement: Supplementary file 1 — Additional file 1. Statement for experiments involving human participants. [file 12860_2022_457_MOESM1_ESM.pdf]

Statement for experiments involving human participants

The informed consent was obtained from all subjects and/or their legal guardian(s). This study was approved by the Ethics Committee of the Fifth Central Hospital of Tianjin.The study was carried out in accordance with the Declaration of Helsinki.
