# Supplementary material for: Overexpression of lncRNA HOXA-AS2 promotes the progression of oral squamous cell carcinoma by mediating SNX5 expression
Source: BMC Mol Cell Biol. 2022 Dec 17;23:59. doi: 10.1186/s12860-022-00457-y (PMC9759889; doi:10.1186/s12860-022-00457-y)

All images for migration and invasion of Fig 2

Figure 2C

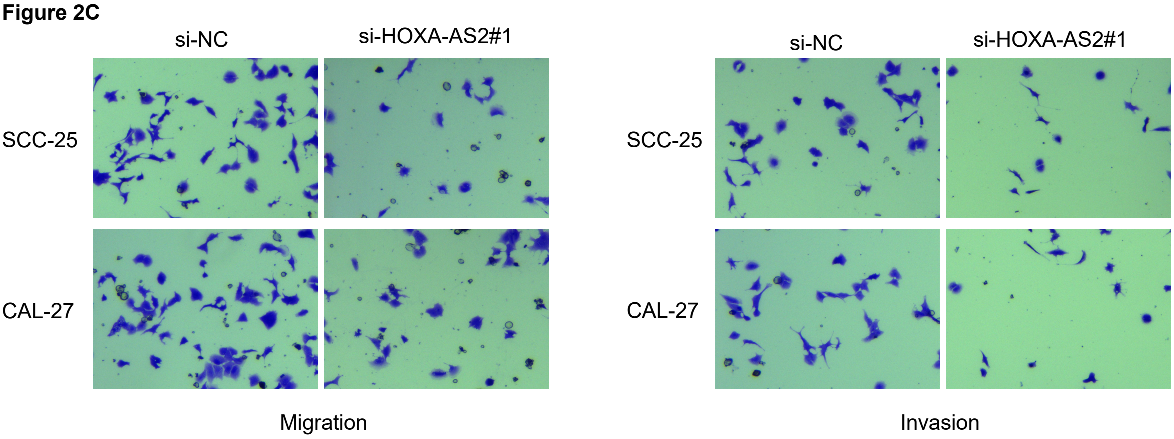

Figure 2D

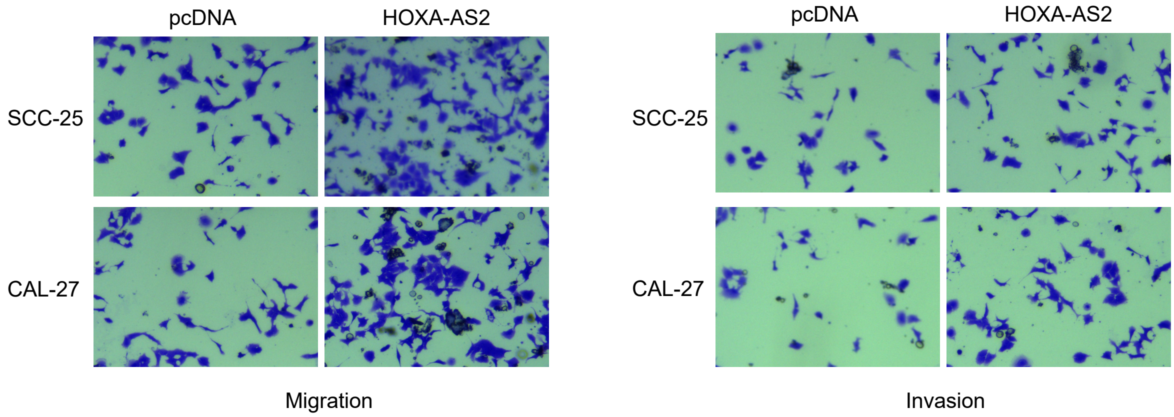

All images for migration and invasion of Fig 3 and Fig 5

Figure 3I

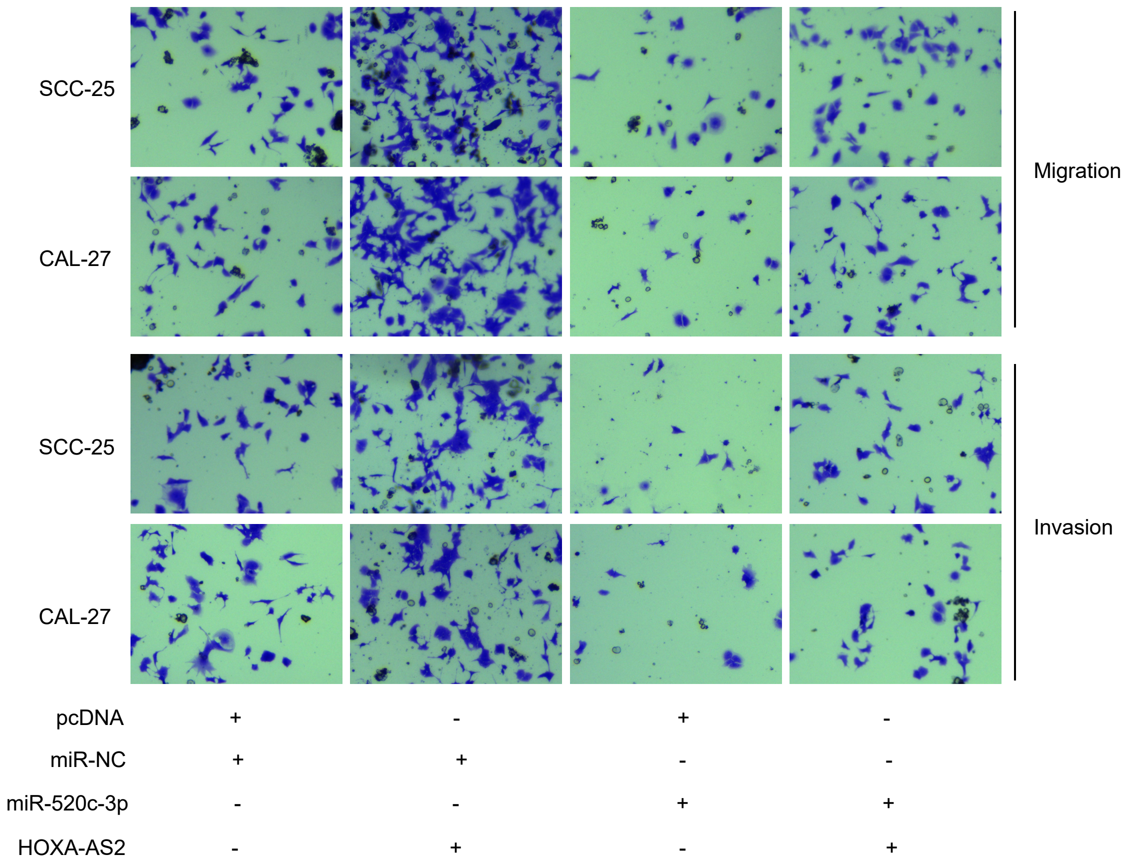

Figure 5F

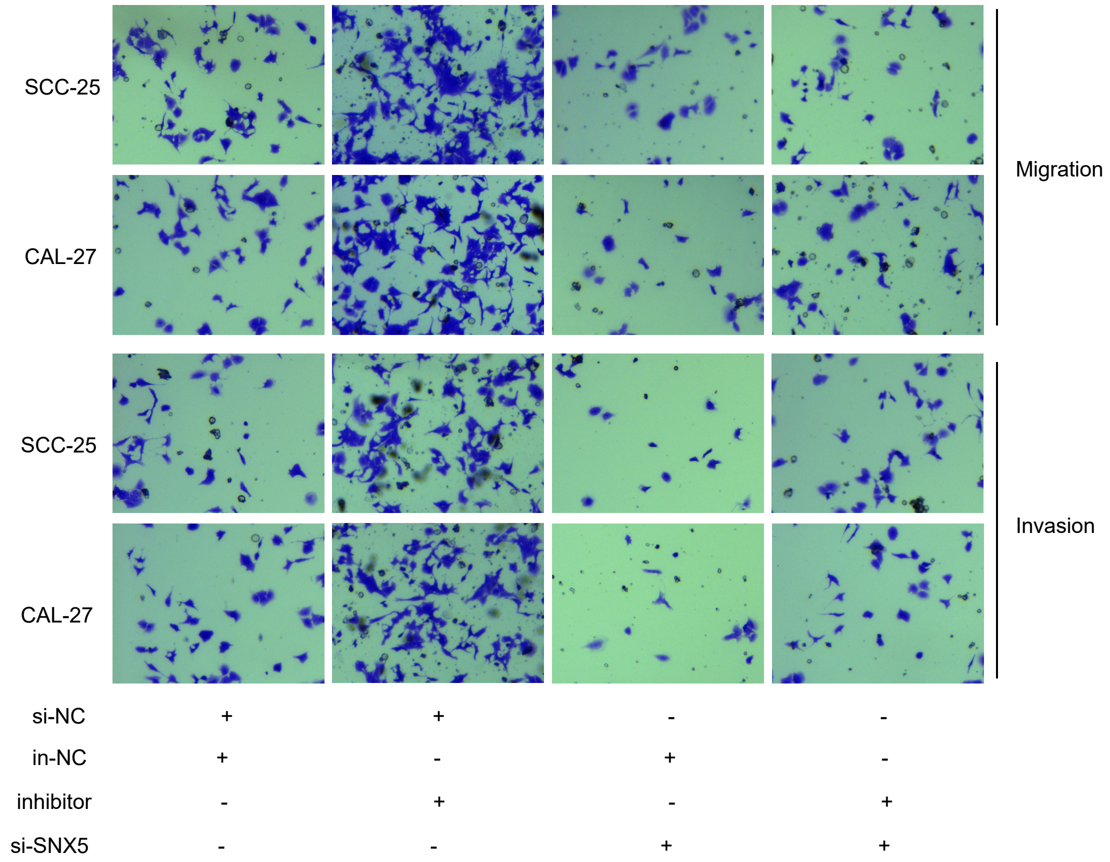

Supplement: Supplementary file 2 — Additional file 2. Supplement Original migration and invasion images. [file 12860_2022_457_MOESM2_ESM.pdf]
