# Supplementary material for: Overexpression of lncRNA HOXA-AS2 promotes the progression of oral squamous cell carcinoma by mediating SNX5 expression
Source: BMC Mol Cell Biol. 2022 Dec 17;23:59. doi: 10.1186/s12860-022-00457-y (PMC9759889; doi:10.1186/s12860-022-00457-y)

All original images of western blots of Fig 5

N1 T1 N2 T2 N3 T3

37 kDa

SNX5

GAPDH

47 kDa

**Figure 5G**

pcDNA

miR-NC

CAL-27

SCC-25

HOXA-AS2

miR-520c-3p

37 kDa

47 kDa

**Figure 5H**

37 kDa

47 kDa

SNX5

GAPDH

miR-NC

HOXA-AS2

miR-520c-3p

pcDNA

SNX5

GAPDH

si-NC+in-NC

si-HOXA-AS2#1+in-NC

si-HOXA-AS2#1+inhibitor

SNX5

GAPDH

37 kDa

47 kDa

si-NC+in-NC

si-HOXA-AS2#1+in-NC

si-HOXA-AS2#1+inhibitor

SNX5

GAPDH

37 kDa

47 kDa

**Figure 5I**

SCC-25

CAL-27


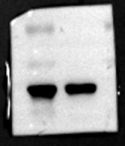

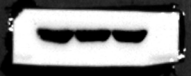

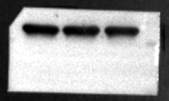

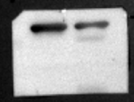

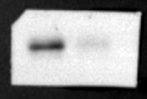

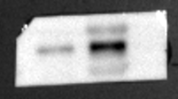

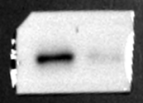

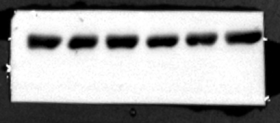

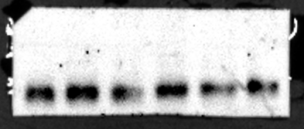

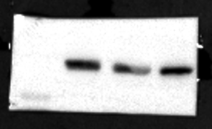

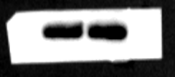

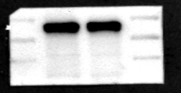

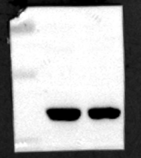

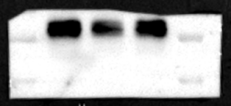


**Figure 6C**

**Figure 6D**

**Figure 6E**

**Figure 6F**

p-mTOR

mTOR

p62

LC3B

Bcl2

Bax

GAPDH

si-NC

si-SNX5

37 kDa

289 kDa

289 kDa

62 kDa

16 kDa

14 kDa

26 kDa

20 kDa

si-NC

si-SNX5

SCC-25

CAL-27

si-SNX5+3MA

si-NC

si-SNX5

si-SNX5+3MA

si-NC

si-SNX5

SCC-25

CAL-27

SCC-25

CAL-27

SCC-25

CAL-27

p-mTOR

mTOR

p62

LC3B

Bcl2

Bax

GAPDH

37 kDa

289 kDa

289 kDa

62 kDa

16 kDa

14 kDa

26 kDa

20 kDa

p-mTOR

mTOR

p62

LC3B

Bcl2

Bax

GAPDH

p-mTOR

mTOR

p62

LC3B

Bcl2

Bax

GAPDH

37 kDa

289 kDa

289 kDa

62 kDa

16 kDa

14 kDa

26 kDa

20 kDa

37 kDa

289 kDa

289 kDa

62 kDa

16 kDa

14 kDa

26 kDa

20 kDa

pcDNA

HOXA-AS2

HOXA-AS2+si-SNX5

HOXA-AS2+miR-520c-3p

pcDNA

HOXA-AS2

HOXA-AS2+si-SNX5

pcDNA

HOXA-AS2

HOXA-AS2+miR-520c-3p

pcDNA

HOXA-AS2

All original images of western blots of Fig 6


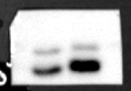

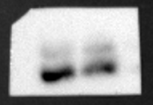

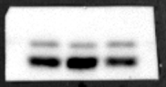

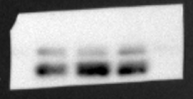

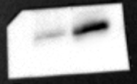

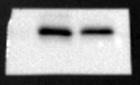

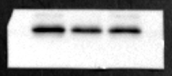

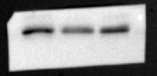

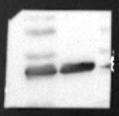

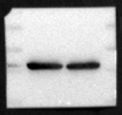

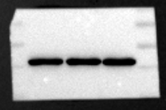

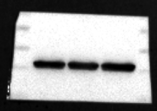

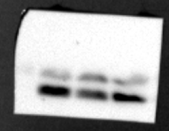

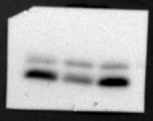

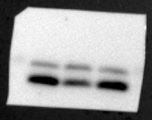

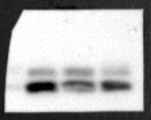

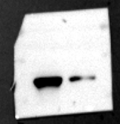

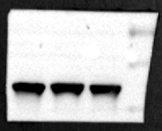

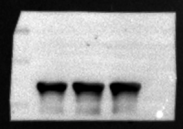

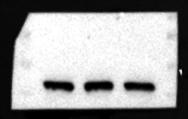

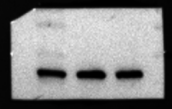

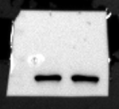

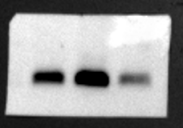

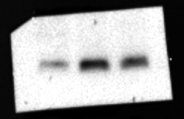

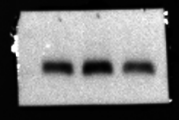

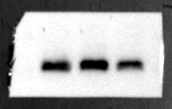

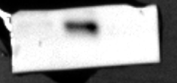

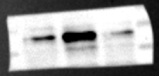

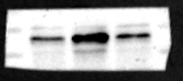

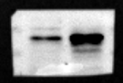

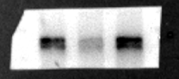

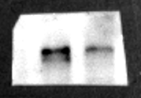

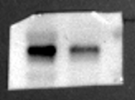

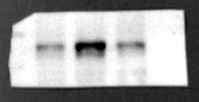

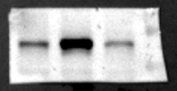

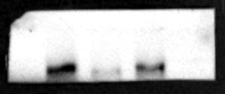

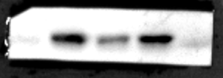

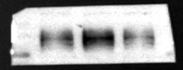

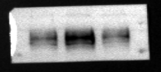

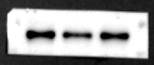

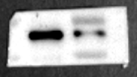

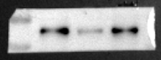

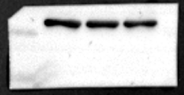

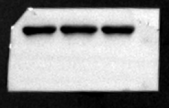

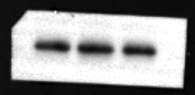

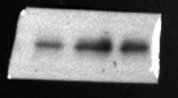

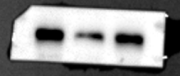

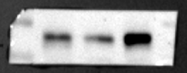

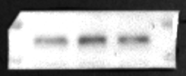

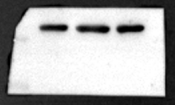

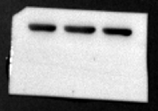

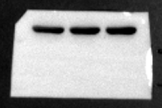

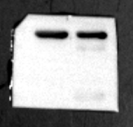

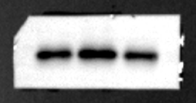

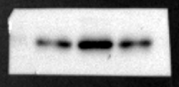

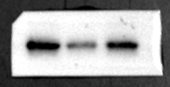

Supplement: Supplementary file 3 — Additional file 3. Supplementary Original western blot images. [file 12860_2022_457_MOESM3_ESM.docx]
